# Defining a role for lung function associated gene *GSTCD* in cell homeostasis

**DOI:** 10.1186/s12931-019-1146-3

**Published:** 2019-08-01

**Authors:** Amanda P. Henry, Kelly Probert, Ceri E. Stewart, Dhruma Thakker, Sangita Bhaker, Sheyda Azimi, Ian P. Hall, Ian Sayers

**Affiliations:** Division of Respiratory Medicine, National Institute for Health Research, Nottingham Biomedical Research Centre, University of Nottingham, Nottingham, UK

**Keywords:** Lung function, GWAS, GSTCD, Epithelium

## Abstract

Genome wide association (GWA) studies have reproducibly identified signals on chromosome 4q24 associated with lung function and COPD. *GSTCD* (Glutathione *S*-transferase C-terminal domain containing) represents a candidate causal gene in this locus, however little is currently known about the function of this protein. We set out to further our understanding of the role of GSTCD in cell functions and homeostasis using multiple molecular and cellular approaches in airway relevant cells. Recombinant expression of human *GSTCD* in conjunction with a GST activity assay did not identify any enzymatic activity for two *GSTCD* isoforms questioning the assignment of this protein to this family of enzymes. Protein structure analyses identified a potential methyltransferase domain contained within GSTCD, with these enzymes linked to cell viability and apoptosis. Targeted knockdown (siRNA) of *GSTCD* in bronchial epithelial cells identified a role for GSTCD in cell viability as proliferation rates were not altered. To provide greater insight we completed transcriptomic analyses on cells with *GSTCD* expression knocked down and identified several differentially expressed genes including those implicated in airway biology; fibrosis e.g. *TGFBR1* and inflammation e.g. *IL6R*. Pathway based transcriptomic analyses identified an over-representation of genes related to adipogenesis which may suggest additional functions for GSTCD. These findings identify potential additional functions for GSTCD in the context of airway biology beyond the hypothesised GST activity and warrant further investigation.

## Background

Lung function forms a key diagnostic criteria for chronic obstructive pulmonary disease (COPD), a leading cause of morbidity and mortality. A greater understanding of genetic variants associated with lung function and COPD may provide new insight into disease mechanisms and provide new opportunities for therapeutic intervention. In 2010, two large consortia for respiratory research: SpiroMeta consortium (*n* = 20,288 individuals of European ancestry) and Cohorts for Heart and Aging Research in Genomic Epidemiology (CHARGE) consortium (*n* = 21,209 individuals of European ancestry) conducted the first in a series of meta-analyses of GWA studies which identified a selection of loci associated with lung function, the most significant of which were novel variants at locus 4q24 [1, 2].

Later studies suggested two independent signals in this region [3, 4] including variants at the *GSTCD* and the *NPNT* gene. Importantly, the genetic effects appear to be independent of both smoking and disease status [2]. More recent studies replicated association with variants spanning *GSTCD* and forced expiratory volume in one second (FEV_1_), forced vital capacity (FVC) and COPD [5–12]. Furthermore, these *variants* have recently been associated with FEV_1_ in children and with growth, measured as bronchial responsiveness development [13], thus suggesting that GSTCD may have an early life effect. Therefore, *GSTCD* represents a potential candidate/casual gene in this region and may functionally contribute to the development or severity of COPD.

In our previous work we demonstrated that *GSTCD* mRNA is expressed in multiple airway relevant cell types and total lung mRNA expression of *GSTCD* correlated with lung function [14]. GSTCD protein expression decreased between the pseudoglandular and canalicular stages in human lung suggesting a potential role in development. Little is known about the function of GSTCD in cells and tissues and based on homology it has been designated a potential member of the Glutathione S-Transferase (GST) family of enzymes due to a shared C-terminal α-helical domain [2]. GST enzymes carry out cellular detoxification, by conjugating glutathione to a variety of endogenous targets [15]. The function of GSTCD particularly in airway cells remain to be determined.

To elucidate the role of GSTCD in cell function and homeostasis we used multiple approaches including; recombinant expression (gain of function) combined with GST activity assessment to define the activity of GSTCD. Similarly, to test the hypothesis that GSTCD is required for normal cell homeostasis we used targeted knockdown (loss of function) in primary human airway epithelium followed by analyses of proliferation, apoptosis and Reactive Oxygen Species (ROS) quantification. Importantly, in parallel we used a hypothesis free approach to identify functions of GSTCD by examining global transcriptomic changes in human bronchial epithelial cells (HBECs) with targeted knockdown of GSTCD.

Overall, we demonstrate that GSTCD does not have GST activity suggesting yet undiscovered functions of this uncharacterized protein. Our findings using multiple approaches suggests a role in primary human cell homeostasis influencing cell viability including also a regulatory function influencing several genes related to airway biology e.g. Transforming growth factor β (*TGFB*) and Interleukin 6 (*IL6*) signalling and genes related to adipogenesis.

## Methods

### GST activity assay

The SensoLyte® GST Activity Assay Kit (AnaSpec) was used to determine the GST activity of cell hosts; CHO-K1, Human airway smooth muscle (HASM) and human bronchial epithelial (HBEC) cells engineered to express GSTCD or GSTM5 (positive control) in 96 well plate format as described by the manufacturer. Cells were maintained in culture as previously described; CHO-K1 [16], HASM [17], HBEC [18]. Briefly, cells were transfected with plasmids pIRES-GSTCDT1, pIRES-GSTCDT2 (two isoforms of GSTCD, variant 1 NP_001026890.2 and GSTCD variant 2 NP_079027.2 where variant 2 contains a truncated exon 2 leading to an 87amino acid shorter protein), pIRES-Empty Vector (EV, negative control) or pcDNA-GSTM5 (positive control) as described [19] and after 48 h transfection the enzymatic reaction was measured on a Flexstation®3 (Molecular Devices).

### Protein prediction modelling of GSTCD

Three protein prediction servers (I-TASSER, SWISS-MODEL and Phyre2). These structure modelling servers were integrated for homology for both GSTCD variants; NP_001026890.2 and NP_079027.2 with proteins of known structure and function. Three primary template based protein structure modelling servers were employed to infer potential 3D protein structures and function of GSTCD based on sequence homology [20] (Iterative Threading Assembly Refinement (I-TASSER) (threading method), SWISS-MODEL and Phyre2 (comparative modelling methods)). Sequences for both published human GSTCD protein variant sequences (NCBI references: GSTCD variant 1 NP_001026890.2 and GSTCD variant 2 NP_079027.2) were input into the servers and analysed by comparing results from the different protein prediction servers alongside results for the positive control Glutathione S-transferase Mu 5 (NCBI reference: NP_000842.2).

### GSTCD SiRNA experiments

HBECs were grown as described [18] and knockdown of GSTCD was achieved by transfection with siRNAs as described [21]. Three pre-designed sequences (A, B and C) targeted to different regions of the *GSTCD* gene (ORIGENE SR312613) were used to reduce expression of GSTCD and a scrambled control siRNA sequence was included for reference. Optimisation of the siRNA concentration transfected into HBECs using INTERFERin® transfection reagent (Polyplus Transfection, 409–10), was carried out using 0.1 nM, 1 nM and 10 nM for each individual SiRNA A, B, or C and a combination of all 3 for 48 h prior to assessing *GSTCD* mRNA levels using qPCR. Two of the 3 siRNAs were chosen to take forward (to provide confidence and account for off target effects) and a time course (12, 24, 48 and 72 h) was used to optimise the reduction in *GSTCD* mRNA. Two of the 3 siRNAs were chosen to take forward (to provide confidence and account for off target effects) at a concentration of 1 nM siRNA. Forty-Eight hours post transfection RNA for Taqman and protein for Western blotting was extracted from these cells as described below. The *GSTCD* Pre-Developed TaqMan® Assay Reagent (PDAR) was chosen across Exons 5 and 6 of *GSTCD* and an *18S* PDAR was used as the housekeeping control for Taqman.

Cell experiments were set up in 6 well culture plates. At the specified time points cells were either used for RNA or protein extraction by the following methods.

The GenElute RNA extraction kit (Sigma) lysis solution was prepared according to protocol with the addition of Beta-Mercaptoethanol. Two wells of a six well plate per condition were washed in ice cold PBS followed by the addition of 600ul lysis solution, cells were scraped off and transferred into an Eppendorf. This was frozen at -80 °C until the RNA extraction was carried out at a later date.

The protein lysis extraction buffer was made up of 1.5 ml unsupplemented cytobuster (cytobuster is one tablet dissolved in 10 ml of cytobuster liquid), 15ul Benzone Nuclease, 1.5ul Dithiothreitol and 3ul Pepstatin A. Cells were washed in ice cold phosphate buffered saline and 200ul of protein lysis buffer was added into one well before cells were scraped off the plastic and this was then transferred into a second well of cells for lysis. This was then placed in an Eppendorf and frozen at -80 °C until the protein was used for quantification and Western blotting. Two polyclonal primary antibodies against GSTCD were used at dilutions of 1:500 and 1:1000 (Proteintech, 17,502–1-AP and Abnova, H00079807-B01) with β-actin antibody (Abcam, ab8227) diluted 1:5000 used as a control in the Western blotting.

### Cell proliferation, number and apoptosis assays

After *GSTCD* siRNA knockdown in HBECs the cell proliferation rate, total cell number and apoptosis were quantified at different time intervals using the CyQUANT® NF proliferation assay, Click-iT® EdU microplate assay and Apoptosis was measured using the In situ cell death detection kit (Roche).

The CyQUANT® NF proliferation assay measures total cell number utilising a cell permeable fluorescent DNA binding dye applied to the cells at the experimental end-point. Click-iT® EdU microplate assay labels actively proliferating cells by incorporating the thymidine analogue 5-ethynyl-2-deoxyuridine (EdU) into DNA during cell replication, followed by antibody based signal amplification resulting in a fluorescence reading. Apoptosis was measured using the In situ cell death detection kit (Roche). In this assay the green fluorescent is an indicator of broken down DNA strands. The HBECs were seeded on 8-well chamber slides and transfected with the siRNA as previously described, cells were then fixed and the ratio of green apoptotic cells to the total number of blue DAPI stained cells were compared.

### RNA sequencing

RNA integrity (RIN) analysis was carried out on the Agilent 2100 Bioanalyser to check quality (RIN > 9). Library preparation and sequencing was performed by Deep Seq, University of Nottingham. Paired end sequencing was performed using the Illumina NextSeq500 and generated 60 million paired end reads (75 bp) for each sample. Analyses of the RNA-seq data used the previously developed pipeline [21] described briefly here. Trimmed reads were aligned to the reference genome (human genome hg19) using TopHat v2.0.12 [22] adapter trimming was achieved using Scythe© (https://github.com/vsbuffalo/scythe) and “Quality Trimming” was performed using Sickle (https://github.com/najoshi/sickle). Reads underwent quality control analysis before and after trimming using FastQC© v0.10.1 (Simon Andrews, Babraham Bioinformatics). Differential Gene Expression Analysis was performed using the Cufflinks suite [22]. Briefly, alignment files were run through Cufflinks for transcriptome assembly. The assemblies were merged using Cuffmerge. Differential expression by Cuffdiff with a false discovery rate (FDR) of 5%. The inclusion of two siRNA targeting GSTCD provided an initial filter of results and we focussed to genes showing differential expression in both siRNA. Finally to provide greater robustness to the RNA-seq differential expression analysis we added a series of further filters including continuity in fragments Per Kilobase of transcript per Million mapped reads (FPKM) values across triplicates, which left genes significantly altered in the *GSTCD* knockdown compared to the scrambled control levels of expression only.

### Gene set enrichment pathway analysis

Using the Gene set Enrichment Analysis (GSEA) from the Molecular signatures database we used the Curated gene set (C2) comprising 4738 gene sets and the Hallmark gene set (H) comprising 50 pathways. An FDR of 25% (FDR q = 0.25) indicates that the result is likely to be valid 3 out of 4 times, which is reasonable in the setting of exploratory discovery where one is interested in finding candidate hypothesis to be further validated as a results of future research [23, 24].

### Reactive oxygen species (ROS) assay

The Oxiselect™ In Vitro ROS Assay Kit (Life Technologies) was used to measure the total free radical activity in supernatants taken from the GSTCD siRNA transfected HBEC experiments. Supernatants were plated in triplicate in 96 well plates and mixed with a catalyst to accelerate the oxidative reaction. A fluorescent probe (DCF-DiOxyQ) was added and the plate analysed on a Flexstation®3, the free radical content is determined by using a standard curve. The experiment was carried out on both fresh and frozen supernatants and fresh homogenised cells.

### Statistical analysis

Data were analysed using GraphPad Prism 6 software (GraphPad Software, Inc.). The Kruskal-Wallis statistic was performed and median and inter-quartile range shown on the graphs. RNA seq differential Gene Expression Analysis was performed using the Cufflinks suite [22]. For all statistical analysis described above, a *p*-value of <0.05 was considered to be significant.

## Results

### GSTCD does not demonstrate classical GST activity

**Fig. 1:**
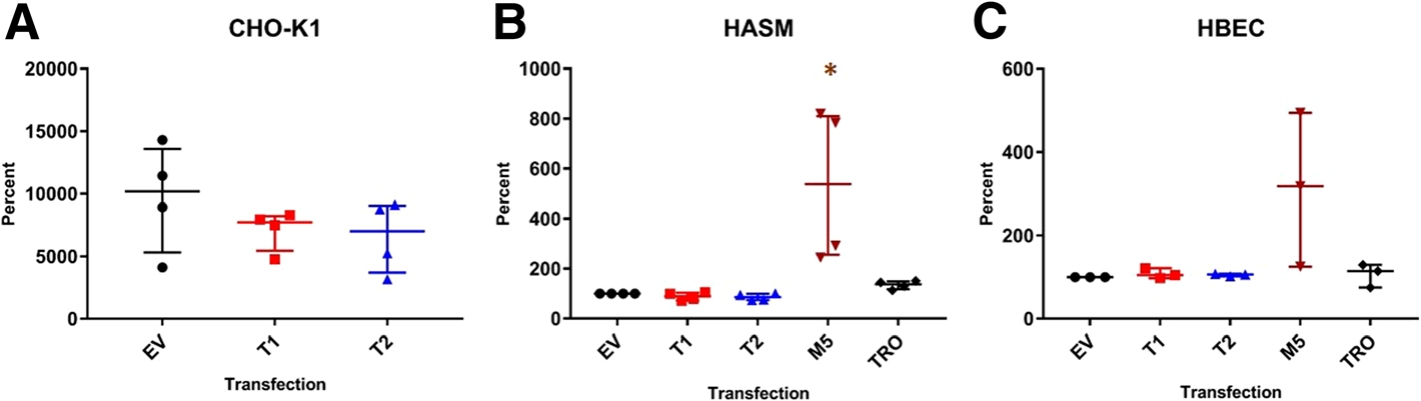
Recombinant expression of GSTCD in multiple cell types does not identify GST activity Levels of GST activity are determined through fluorescence measurements (mU/ml) and expression is shown relative to EV. Graphs show median and interquartile range data analysed using Kruskal-Wallis test. **a** GST activity in CHO-K1 cells stably transfected with GSTCD variant 1 (T1), GSTCD variant 2 (T2) or empty vector (EV) showing no increased activity above the EV control (*n* = 4). **b** HASM cells transiently transfected with EV control, T1, T2, GSTM5 positive control (M5) and transfection reagent only (TRO). The relative GST activity was shown to be significant between the EV and M5 (*p* = 0.002,) however the two isoforms of GSTCD did not show any increased GST activity (*n* = 4). **c** HBEC cells transliently transfected with the plasmids showed no significant increases in GST activity over the EV (*n* = 4)

We expressed recombinant *GSTCD* in a number of cell hosts including CHO-K1, HASM and HBEC to determine if the GSTCD protein had GST activity. Two vectors expressing different isoforms of human GSTCD were transfected into two human airway cell types: airway smooth muscle (HASM) and bronchial epithelial cells (HBECs) alongside transfection of the positive control GST Mu5 (GSTM5). CHO-K1 which have endogenous GST activity was also transfected with the *GSTCD* plasmids. The two GSTCD isoforms differ in exon 2 (variant 2 is truncated) resulting in proteins of 633 amino acids and 546 amino acids respectively for variant 1 and 2 [14]. The Sensolyte GST activity assay was performed on these recombinant cells, results revealed no discernible change in GST activity in either GSTCD variant 1 or 2 cells (relative to empty vector and transfection reagent only negative controls) (Fig. 1). In contrast, recombinant GSTM5 cells, a well characterised GST enzyme resulted in 2–3 fold increased GST activity relative to the negative controls (*p* = 0.02 in HASM). Based on these data the two reported protein isoforms of GSTCD do not have atypical GST activity in three independent cell hosts.

### GSTCD protein structure prediction and nucleotide homology searches suggest additional protein functions

To address the lack of information regarding potential GSTCD function, homologous protein sequences were sought using three protein prediction servers I-TASSER, SWISS-MODEL and Phyre2. Homologous matches to the GSTCD protein sequence were searched with results indicating a high homology to methyltransferase enzymes across the servers (Table 1). Sequence homology is predominantly located across the predicted methyltransferase domain (variant 1: 423-541aa and variant 2: 336-454aa) as opposed to the GST C-terminal α-helical domain (variant 1: 251-332aa and variant 2: 164-245aa) and this is consistent throughout the top homologous matches for each server. Therefore these data provide initial insight that GSTCD may have additional protein functions, with methyltransferase enzymes linked to cell viability and apoptosis.

**Table 1:** Protein structure prediction results for the two GSTCD isoforms using three models I-TASSER, SWISS- MODEL and Phyre2. Methyltransferase (MTF) enzymes are frequently found in the top 3 structural templates listed for each server and the top 50 template results in SWISS-MODEL were entirely methyltransferase proteins, with specific focus on rRNA methyltransferase function. Alternatively, I-TASSER identified homology in this region with transport receptors, specifically importin β subunit 1, involved in transporting proteins into the nucleus [25]. Other top hits included: exodeoxyribonuclease (catalyses degradation of double stranded DNA), Ras guanyl-releasing protein 1 (nucleotide exchange factor specifically activating Ras, activates the extracellular signal-regulated kinases/mitogen-activated protein kinase (ERK/MAPK) cascade) and nicotinamide adenine dinucleotide binding (NADB)-Rossmann fold superfamily protein (structural motif found in proteins that bind nucleotides)

Protein Structure Results	I-TASSER		SWISS-MODEL		Phyre2	
	Variant 1	Variant 2	Variant 1	Variant 2	Variant 1	Variant 2
Predicted Secondary Structure	Primarily α-helices with intermittent coil structures. β-strands only present at termini.		N/A		Primarily intermittent α-helices (45%). β-strands only present at termini (12%). Two predicted transmembrane regions (30-40aa and 281-299aa).	Primarily intermittent α-helices (55%). β-strands only present at termini (10%). Two predicted transmembrane regions (29-38aa and 194-212aa).
Regions of Primary Sequence Homology	Primarily across the MTF domain to C-terminus. Partial matches across whole protein.		Only across the MTF domain to C-terminus		Only across the MTF domain	
Intrinsic disorder	N/A		Low disorder across C-terminus	High disorder across C-terminus	Two theoretical central regions of disorder (27%):• 204-270aa• 325-386aa	Two theoretical central regions of disorder (26%):• 118-184aa• 257-299aa
Top 3 Structural Templates	Putative MTF Importin β subunit Exodeoxyribonuclease	Ras guanyl-releasing protein 1 MTF	Protein RdmB MTF Protein RdmB MTF Protein RdmB MTF	Putative rRNA methylase Putative rRNA methylase rRNA small subunit MTF	rRNA small subunit MTF Transferase S-adenosyl-L-methionine-dependent MTF	Transferase NADB-Rossmann fold superfamily protein S-adenosyl-L-methionine-dependent MTF

### Targeting GSTCD by RNAi significantly attenuates both mRNA and protein expression in primary bronchial epithelial cells

**Fig. 2:**
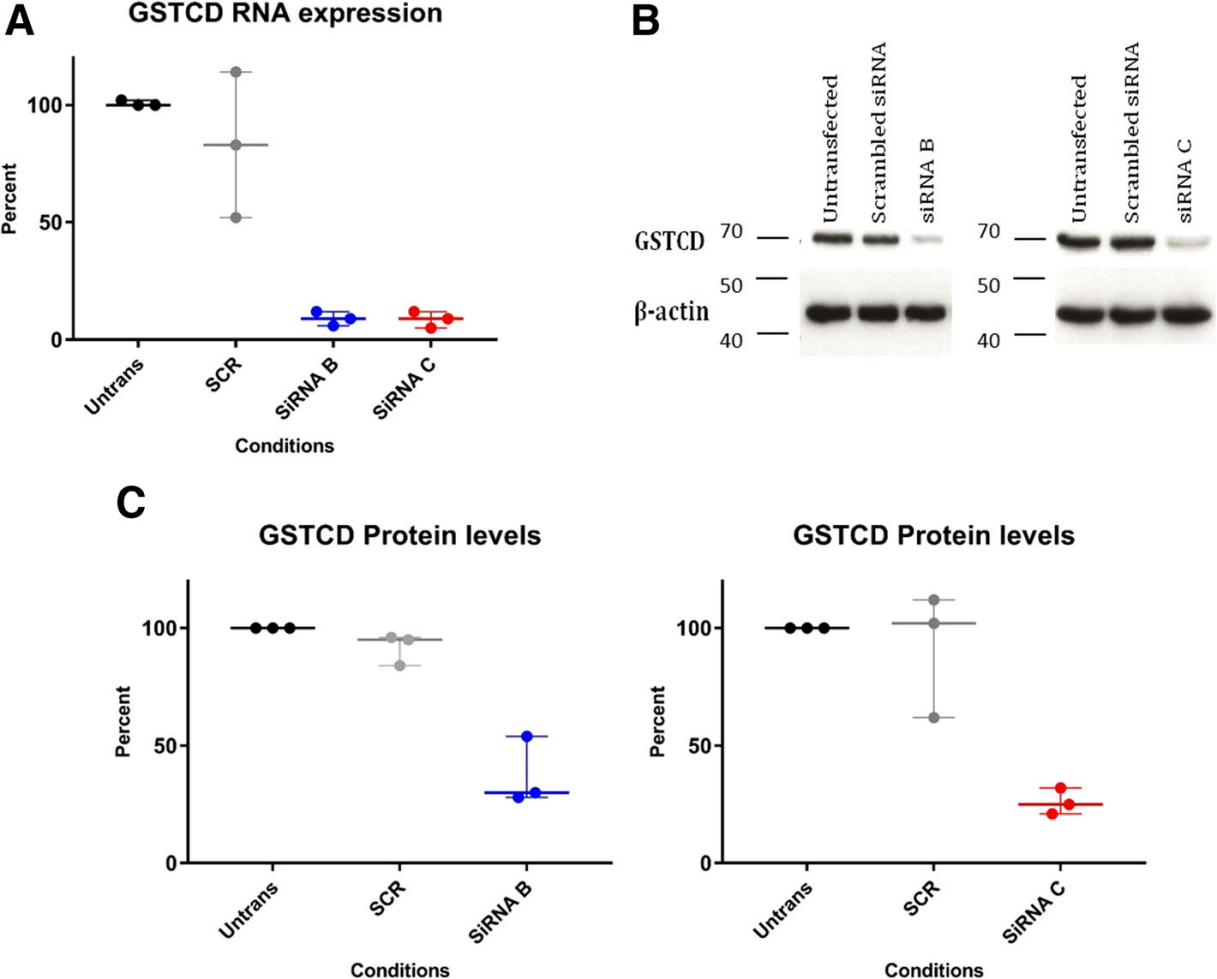
GSTCD can effectively be targeted in vitro leading to a reduction in mRNA and protein levels. **a**. Shows RNA expression using taqman normalised to the untransfected condition. Both SiRNA B and C knocked down the GSTCD RNA to a similar extent. **b**. Western blots to show the GSTCD protein levels (71 kDa) are reduced in hBEC lysates following siRNA knock-down. GSTCD protein levels were noticeably reduced relative to untransfected and scrambled controls for each siRNA in all samples (*n* = 3). **c**. Semi-quantification of GSTCD protein levels by densitometry. Proportional changes are shown with results normalised against untransfected control. GSTCD protein expression is significantly reduced for siRNA B (*p* < 0.0036) and siRNA C (*p* < 0.036) using Kruskal Wallis test showing median and interquartile range. *N* = 3 independent experiments

To begin to understand the role of GSTCD in the cell we optimised GSTCD knock down in HBEC using the siRNA technique. Optimal conditions for maximal knock down were 1 nM siRNA incubated for 48 h and resulted in 88% (siRNA B) and 90% (C) decrease in GSTCD mRNA (Fig. 2), with 86% (siRNA A, data not shown). SiRNA B and C were taken forward in further experiments to help distinguish between true and off target effects.

Semi-quantified Western blotting focussed to experiments using the siRNA B and C demonstrated GSTCD expression was reduced by 63% (siRNA B) and 74% (siRNA C) relative to untransfected control lysates (siRNA B *p* < 0.0036 and C *p* < 0.036, Kruskal Wallis. Figure 2). Therefore, both siRNA B and siRNA C effectively reduced GSTCD mRNA and protein expression in HBECs providing a platform for functional analyses.

### Targeted GSTCD knock down does not influence cell proliferation but is associated with reduced numbers of bronchial epithelial cells when cultured over 72 h

**Fig. 3:**
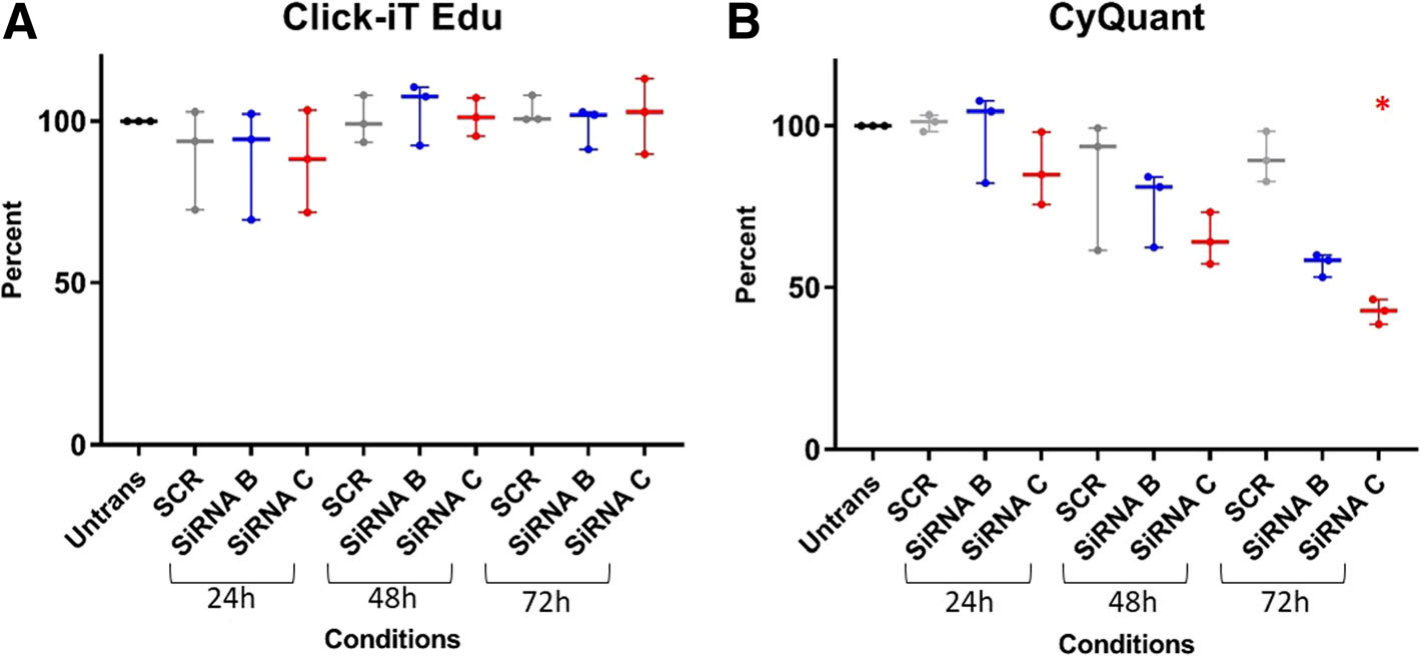
GSTCD siRNA knock-down in hBECs results in reduced total cell number while not influencing proliferation. GSTCD siRNA knock-down in hBECs does not affect proliferation rate (Click-iT® EdU) but does result in reduced total cell number over 72 h (CyQuant) in cell culture. Graphs show median and interquartile range data analysed using Kruskal-Wallis test. **a**. The Click-iT® EdU assay data is normalised to Untransfected controls (100%) bar. No change in proliferation rate was seen in GSTCD knock-down samples (siRNA B and C) relative to untransfected or scrambled controls at any time-point. **b**. The CyQuant assay shows a noticeably reduced total cell number in both GSTCD knock-down samples, with an increasing effect across the time-course and significant reduction evident at 72 h compared to scrambled for siRNA C (*p* = 0.02).

To assess potential effects of GSTCD knock-down on cell growth two assays were utilised to investigate actively proliferating cells (Click-iT® EdU assay) and total cell number (CyQUANT® NF assay). GSTCD expression was knocked-down in HBECs as previously described over a 24, 48 and 72 h. No discernible changes in levels of actively proliferating cells were observed in either siRNA GSTCD knock-down sample relative to untransfected or scrambled controls (Fig. 3a). However total cell number was reduced in both siRNA B and C across time relative to untransfected or scrambled siRNA treated hBECs, with siRNA C displaying the greatest magnitude of effect (Fig. 3b). At 72 h total cell number was significantly reduced compared to scrambled siRNA for siRNA C (*p* < 0.02) when results are normalised to untransfected cells.

### Targeted GSTCD knockdown does not have any impacts on apoptosis rate in bronchial epithelial cells

**Fig. 4:**
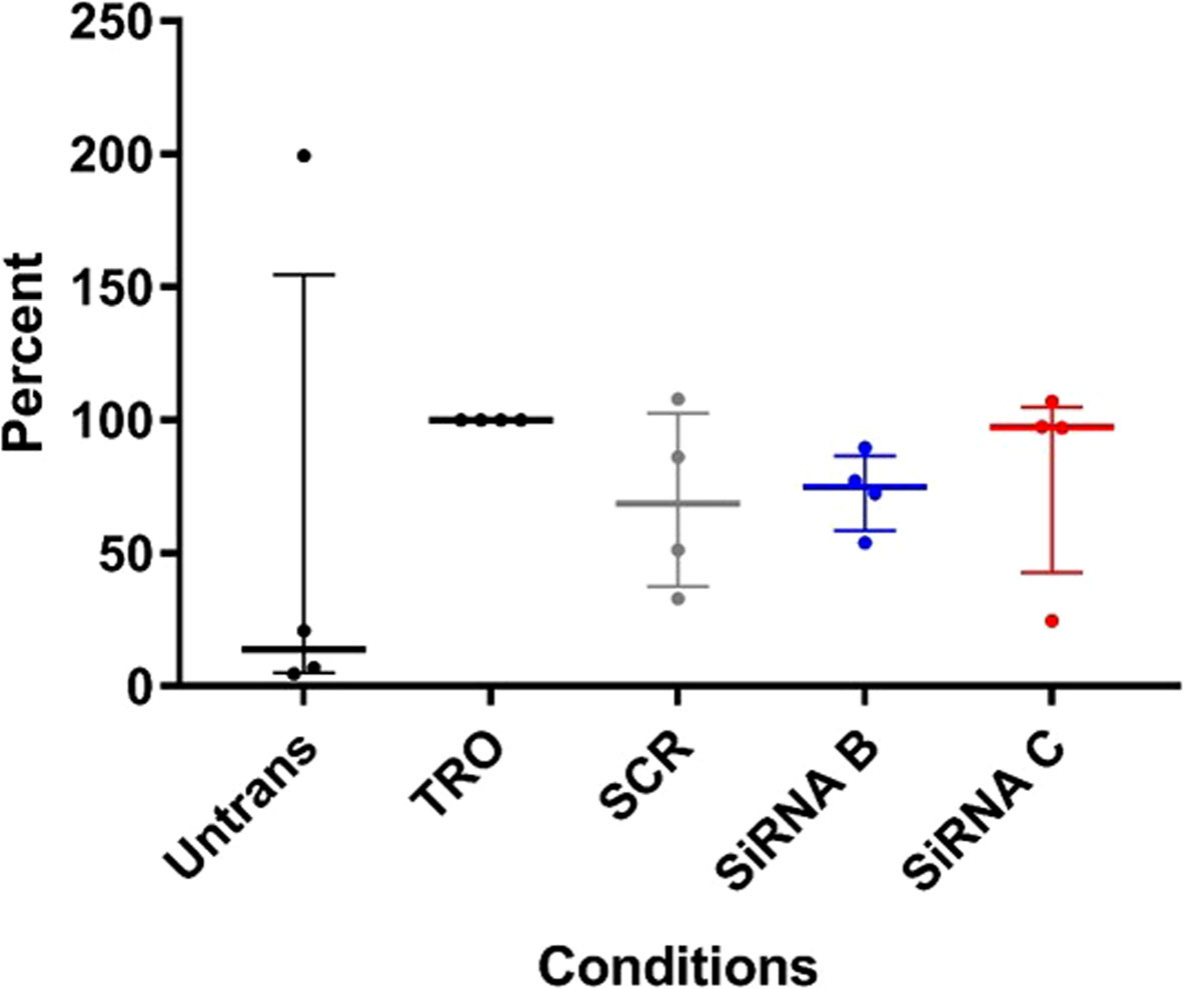
GSTCD siRNA knock-down in hBECs does not influence cell apoptosis. The ratio between the Apoptosis assay (Apo-ONE) and the total cell number assay (CyQuant) was used to determine the level of apoptosis in the GSTCD siRNA knock-down conditions taking into account any cell number variability. GSTCD knock down did not influence the level of apoptosis when corrected for cell number in these experiments. TRO, transfection reagent only. No significance using Kruskal Wallis test showing median and interquartile range (*n* = 4)

We wanted to determine if the reduction in total cell number observed in the human cell system could be explained by increased apoptosis in the GSTCD knockout cells. Figure 4 shows that there is no statistically significant difference in apoptosis levels between cells with GSTCD knocked down and the untransfected, transfection reagent alone or scrambled siRNA treated cells. There was a trend toward increase in apoptosis in siRNA B > siRNA C which inversely correlates with the effects of these siRNA on cell numbers. However, overall these data suggest decreased GSTCD expression has no significant effect on apoptosis in airway epithelial cells.

### Targeted GSTCD knockdown in combination with transcriptomics identifies novel potential functions in bronchial epithelial cells

**Fig. 5:**
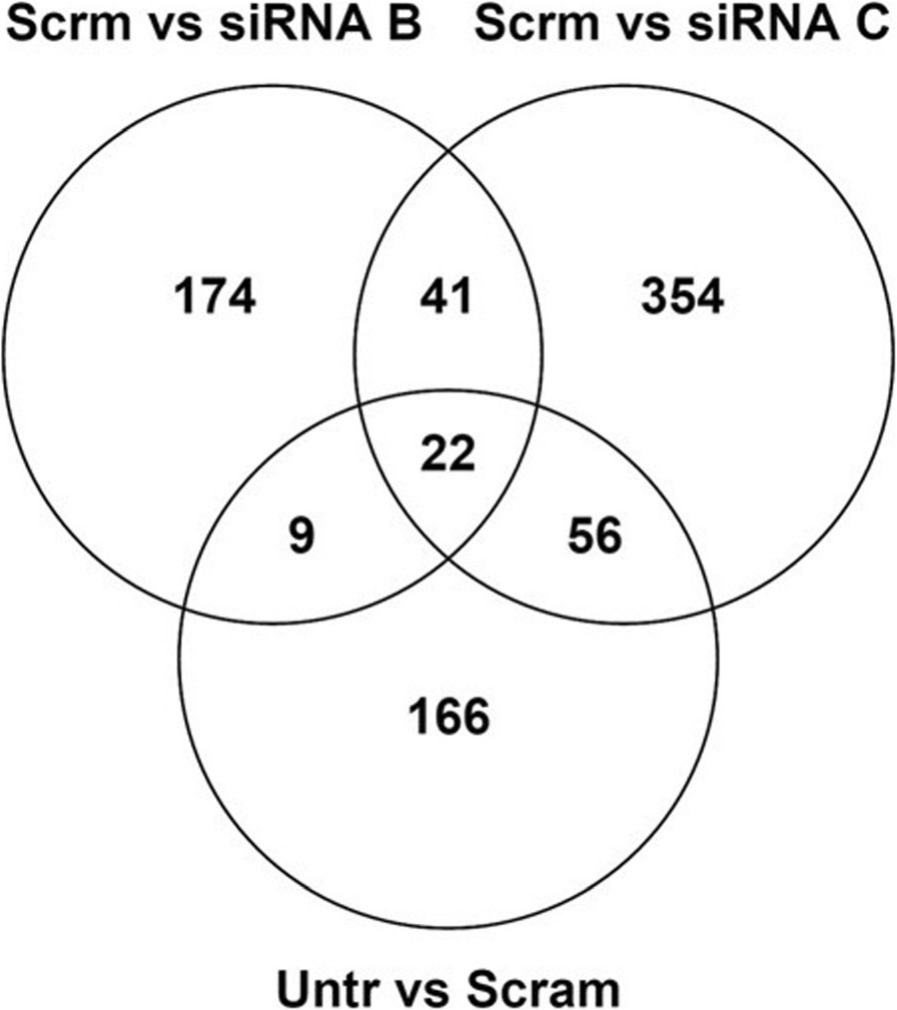
Venn diagram illustrating the number of genes differentially expressed following GSTCD knockdown for the indicated conditions. The GSTCD knock down gene expression profile was compared with the scrambled and shows 41 genes to be reproducibly changed in both siRNA B and C and not confounded by differences between control conditions untransfected/scrambled

In order to identify novel functions of GSTCD in the cells and new understanding we completed a series of transcriptomic experiments using bronchial epithelial cells with GSTCD targeted knockdown. The RNA-seq profiling gave over 50 million aligned paired reads per sample to the hg19 genome build. A FDR of 5% was applied which identified 41 genes showed significant differential mRNA expression levels following *GSTCD* knockdown using either siRNA B or siRNA C compared with scrambled and untransfected gene expression providing an initial focus (Fig. 5). Further refinement of differentially expressed genes to reproducible effects across each biological replicate identified 17 priority genes altered transcription post *GSTCD* knockdown compared to the scrambled control levels (Table 2).

**Table 2:** Shows the list of 17 genes with significant differential expression after GSTCD knockdown with an FDR less than 5% from RNA seq analysis. The * denotes those genes with an expression level similar or higher than GSTCD expression

Gene	Direction of Effect	SCR vs B log2(fold_change)	SCR vs B q_value	SCR vs C log2(fold_change)	SCR vs C q_value
GSTCD	–	2.50688	0.00625	2.60049	0.00306
AKAP12*	–	2.06957	0.00625	1.45912	0.00306
C12orf49*	–	1.41126	0.00625	0.856333	0.00306
CUX2*	–	1.30553	0.00625	0.831766	0.00306
DESI2*	–	1.36908	0.00625	0.69109	0.00306
UHMK1*	–	1.10051	0.00625	1.07772	0.00306
ST6GALNAC1*	+	1.05294	0.00625	1.02191	0.00306
PPAP2C	+	1.05893	0.00625	1.25417	0.00306
XLOC_020899	–	1.54684	0.00625	1.13877	0.01732
TGFBR1*	–	0.86861	0.00625	0.552792	0.04536
GPD1L	–	1.04006	0.01063	1.23236	0.00306
FAT2*	+	0.761438	0.01451	1.045	0.00306
LCP1	–	1.42681	0.01451	1.239	0.03647
TSPAN7	+	1.14222	0.01451	0.915029	0.04412
ETS1*	–	0.672957	0.03997	0.678173	0.01232
GPR176*	–	0.864392	0.04704	0.694795	0.02858
IL6R*	+	0.750533	0.04874	0.825264	0.00306

**Fig. 6:**
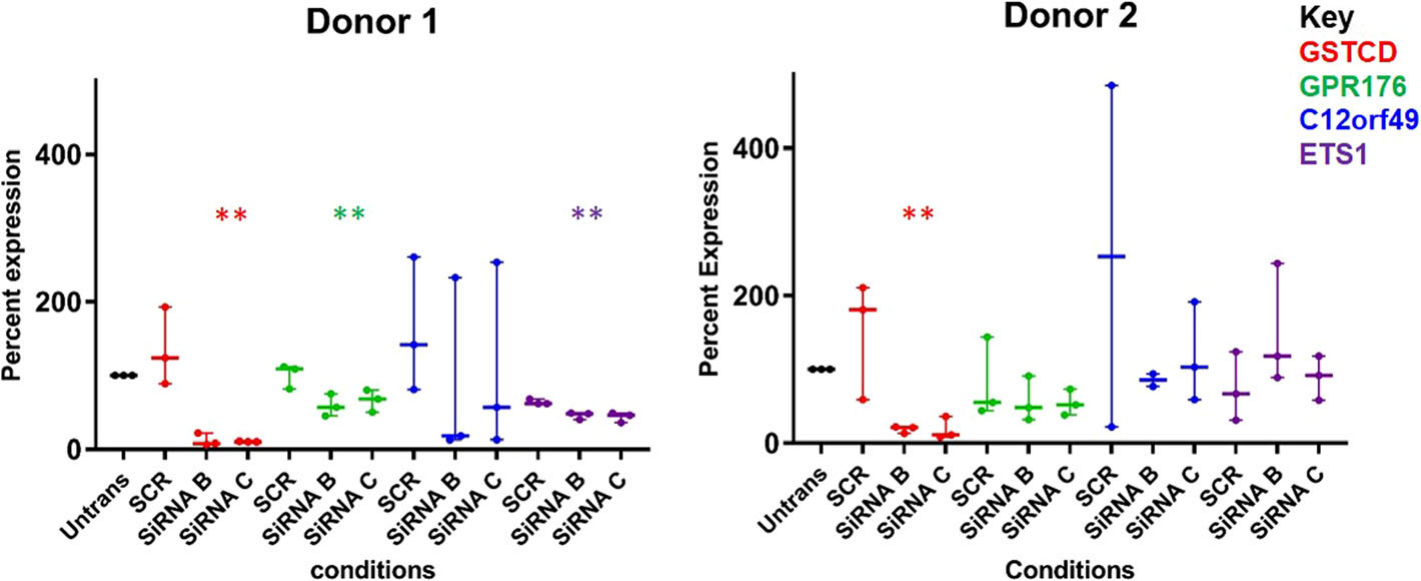
Quantitative PCR analysis of three genes which were differentially expressed after GSTCD knock down in RNA-seq analyses. The Taqman was performed in two different donors, the original donor which RNAseq was performed (donor 1) and a second donor for comparison (donor 2). Untransfected set at 100% expression, red is GSTCD, green is GPR176, blue is C12orf49 and purple is ETS1. The 3 genes were chosen due to a good level of expression before and after GSTCD knock down as well as being high on the RNAseq gene list so that the effect can be seen by taqman analysis. Graph shows median and interquartile range data analysed using Kruskal-Wallis test. In donor 1 there is significant knockdown for both SiRNA B and C in genes GSTCD, GPR176 and ETS1 (*P* < 0.01), in donor 2 only GSTCD reached statistical significance

Reassuringly, *GSTCD* was the most significantly reduced mRNA, then the gene with the next greatest magnitude of change was; Lymphocyte cytosolic protein 1 (*LCP1*) with a 27 and 17 fold induction in mRNA in siRNA B and C respectively. In this modest number of genes identified several have been previously associated with airway biology and disease including e.g. TGFBR1, the receptor for TGFB_1_ involved in fibrosis and IL6R a cytokine receptor implicated in airway disease e.g. asthma. A description of these top 17 genes can be found in Table 3. To provide further reassurance of our findings we investigated the mRNA levels of three abundant mRNA in the original HBEC donor used for RNA seq analysis and also in a second independent donor. The three genes selected were; *GPR176, C12orf49* and *ETS1* (Fig. 6). In the original donor used for the RNA seq experiments taqman significantly confirmed knockdown of expression in GPR176 and ETS1 (*p* = 0.0087 and *p* = 0.0016 respectively Kruskal-Wallis) but was not significant for the C12orf49 gene expression. In the second donor none of these genes showed a significant differential gene expression although the GSTCD expression by taqman was significant for both donor1 and donor2 (*p* = 0.009 and p = 0.008 respectively). This lack of replication in the second donor may be due to the increased heterogeneity observed in this dataset.

**Table 3:** Shows the differentially expressed genes and a description of their possible functions. Noting the expression of mRNA and protein in the lung using GTex data for Median Tags per Million (TPM) and protein atlas expression where data was available

Gene	Gene name	Expression in the lung	Literature search
GSTCD	Glutathione S-transferase C-terminal domain containing	mRNA: 3 TPM Protein: Medium	
LCP1	Lymphocyte cytosolic protein 1	mRNA: 121 TPM Protein: High	Plastins are Actin binding proteins, L-plastin has been found in many types of malignant human cells of non-hemopoietic origin suggesting that its expression is induced accompanying tumorigenesis in solid tissues. It also has 2 calcium-binding domains and a calmodulin-binding domain. GWAS identified this gene in nonalcoholic fatty liver disease. mRNA 300% increase in biopsies from patients with NAFLD [26].
GPR176	G protein-coupled receptor 176	mRNA: 10 TPM Protein: Medium	Gz-linked orphan G-protein coupled receptor. Gpr176 is expressed in a circadian manner by SCN neurons in the brain, and molecular characterization reveals that it represses cAMP signalling in an agonist-independent manner [27].
XLOC_020899	–		
C12orf49	C12 open reading frame 49	mRNA: Yes 34 TPM Protein: Medium	Is a protein found to be expressed mainly in the thyroid and lung.
ETS1	V-ets erythroblastosis virus E26 oncogene homolog 1	mRNA: Yes 91 TPM Protein: None	ETS is a transcription factor for the activation or repression of numerous genes involved in stem cell development, cell senescence and death and tumorigenesis. Meta-analysis GWAS of self-reported allergy [28, 29].
GPD1L	Glycerol-3-phosphate dehydrogenase1-like	mRNA: Yes 37 TPM Protein: N/A	A protein found in the cytoplasm associated with the plasma membrane where it binds to the sodium channel SCN5A.
AKAP12	A kinase (PRKA) anchor protein 12	mRNA: Yes 66 TPM Protein: Low	This protein directs the activity of protein kinase A by tethering the enzyme near its physiologic substrates at the cell periphery. It is a cell growth-related protein.
CUX2	CUT-LIKE 2	mRNA: None Protein: None	Contains 4 DNA binding domains, 3 cut repeats and a homeodomain which also bind DNA. Possibly binding to promotor region of Ncam gene. CUX2 functions as an accessory factor that stimulates the repair of oxidative DNA damage. GWAS associated a SNP in the MYL2-CUX2 region with Gout [30]. These genes are associated with cholesterol and diabetes.
DESI2	DESUMOYLATING ISOPEPTIDASE 2	mRNA: Yes 32 TPM Protein: N/A	Is predicted to have a papain-like fold and to function as a cysteine protease that removes SUMO from SUMO-modified proteins (SUMO small ubiquitin-like modifier). Is regulated in prostate metastasis [31].
UHMK1	U2AF HOMOLOGY MOTIF KINASE 1	mRNA: Yes 28 TPM Protein: N/A	A serine/threonine protein kinase that promotes cell cycle progression through G1 by phosphorylation of the cyclin dependent kinase inhibitor 1B. It is also thought to function in the adult nervous system and the gene has been associated with schizophrenia. Whole genome analysis revealed a gene fusion between *UHMK1* and *DDR2 In well-differentiated liposarcoma* [32].
IL6R	Interleukin 6 receptor	mRNA: Yes 30 TPM Protein: N/A	The receptor of IL6 which is involved in the regulation of the immune response. Has been identified in many studies, SNPS in this gene show altered regulation in airway diseases such as asthma and COPD [33].
FAT2	FAT TUMOR SUPPRESSOR, DROSOPHILA, HOMOLOG OF, 2	mRNA: None Protein: None	This tumour suppressor has 34 tandem cadherin-type motifs, 2 EGF domains, a laminin G domain, a transmembrane domain, a cytoplasmic proline rich region and a cytoplasmic RGD motif. Most likely a cell adhesion molecule controlling cell proliferation. Downregulated in A549 stable tumor cell line, obtained from human lung carcinoma upon exposure to Cobalt [34].
PPAP2C	PHOSPHATIDIC ACID PHOSPHATASE TYPE 2C	mRNA: Yes 9 TPM Protein: N/A	Dephosphorylates Phosphatidic acid to form diacylglycerol. It has roles in metabolic pathways controlling glycerophospholipids and triacylglycerols, and in receptor-activated signal transduction mediated by phospholipase D.
TGFBR1	Transforming growth factor, beta receptor type 1	mRNA: Yes 25 TPM Protein: Medium	A serine/threonine kinase receptor for TGFB1. The type 1 receptor mediates induction of several genes involved in cell-matrix interactions. A mutation in this gene was identified in some patients with Thoracic Aortic aneurysm [35]. An miR-18a-5p inhibits pulmonary fibrosis by targeting TGFB receptors [36].
TSPAN7	Tetraspanin 7	mRNA: Yes 69 TPM Protein: None	This protein is a cell surface glycoprotein known to complex with integrins and a member of the transmembrane 4 superfamily. May have a role in control of neurite outgrowth.
ST6GALNAC1	ST6 ALPHA-N-ACETYL-NEURAMINYL-2,3-BETA-GALACTOSYL-1,3-N-ACETYLGALACTOSAMINIDE ALPHA-2,6-SIALYLTRANSFERASE 1	mRNA: Yes 6 TPM Protein: High	This protein transfers a sialic acid to O-linked GalNAc residues. Usin+A15:D19g the genome-wide promoter activity profiles a novel marker candidate, ST6GALNAC1 for adenocarcinoma of the lung [37] was identified.

### Pathway based analyses of transcriptomic data implicate a role for GSTCD in multiple pathways including; oxidative phosphorylation/reactive oxygen species pathways and adipogenesis

Using the Gene set Enrichment Analysis (GSEA) from the Molecular signatures database we searched the Curated gene set (C2) and the Hallmark gene set (H) pathways. These analyses identified 29 significantly upregulated pathways which were mainly Cancer focussed but also Adipogenesis related and a Reactive Oxygen species (ROS) pathway involved in insulin resistance. Eight pathways were significantly upregulated in the H pathway analysis and no pathway showed any significant downregulation at this 25% FDR (q-value < 0.25) significance level. Table 4 shows results from the H pathway analysis with the number of genes in each pathway and the percent of those genes upregulated in the knockdown cells. Adipogenesis and ROS pathways were significantly upregulated (FDR 25%).

**Table 4:** Summary table of the pathways significantly upregulated in the Hallmark GSEA. The FDR 25% was the significance level cut off (FDR q < 0.25) only upregulated pathways were significant in both GSTCD SiRNA B and SiRNA C gene lists and there were no significantly different pathways with an FDR below 10%

Upregulated Pathways	Number of genes in pathway	Number of Significant genes for B and C (%)	B FDR q-val	C FDR q-val
Adipogenesis	196	46 (23)	0.013	0.012
Bile Acid Metabolism	112	15 (13)	0.1	0.061
Estrogen Response early	199	34 (17)	0.16	0.00081
Fatty Acid Metabolism	158	24 (15)	0.16	0.045
Oxidative Phosporylation	199	62 (31)	0.037	0
P53 Pathway	199	46 (23)	0.1	0.0007
Peroxisome	103	29 (28)	0.2	0.038
Reactive Oxygen Species Pathway	47	12 (25)	0.1	0.061

### GSTCD knockdown and ROS handling in bronchial epithelial cells in vitro

**Fig. 7:**
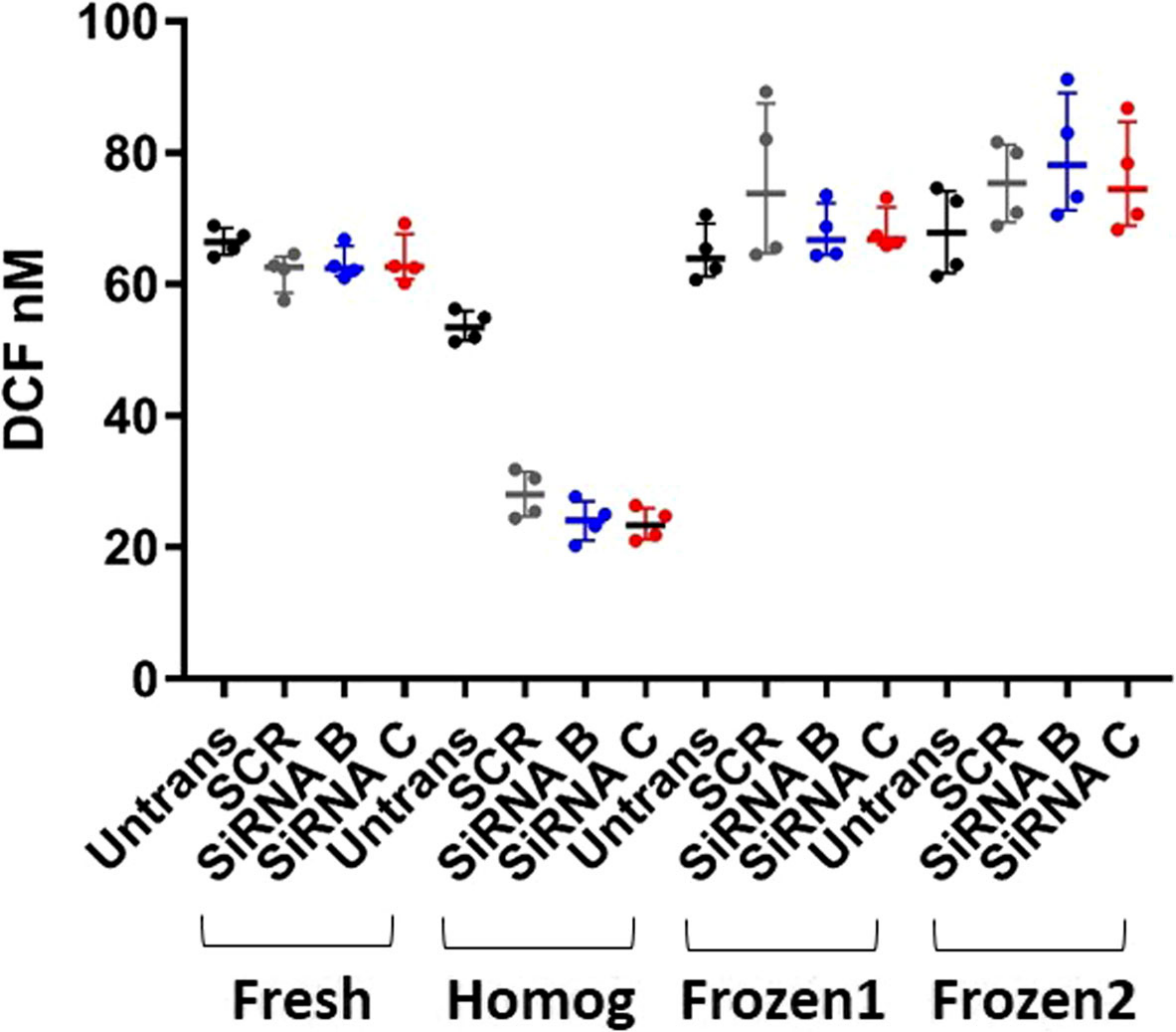
Shows the results of the ROS assay in both fresh and frozen cells and supernatants. No difference was observed in ROS accumulation between the Untransfected, scrambled, GSTCD SiRNA B or C in any of the condition which were: Fresh - Fresh supernatant, Homog – Fresh homogenised cells, Frozen1 and 2 - supernatants stored at -80 °C. This is *n* = 1 from one biological experiment using different cell compartments and conditions for ROS detection

Based on the initial observation that the reactive oxygen species pathway is upregulated in human bronchial epithelial cells with targeted GSTCD based on RNA-seq data we wanted establish whether this knockdown could alter the ROS activity. To do this we used the Oxiselect™ ROS activity assay. Supernatants from fresh, frozen and directly from cells homogenised after 48 h transfection with the GSTCD siRNA were analysed (Fig. 7, no statistics were performed as it was n = 1 for each condition). There was no difference in ROS activity in either the cell supernatants or the cell homogenates from cells with or without GSTCD knockdown.

## Discussion

We set out to further our basic understanding of the still largely uncharacterised protein, GSTCD in the context of a potential role in the airways as genetic variants spanning GSTCD have reproducibly been associated with lung function and COPD in multiple GWAS. To achieve this, we used overlapping and complementary approaches including producing the recombinant protein, targeting GSTCD by siRNA followed by cell biology outcomes and transcriptomic analyses to identify novel functions. Overall we demonstrate that GSTCD does not have measurable GST activity suggesting additional unidentified protein functions, that GSTCD is required for cell homeostasis with reduction of the protein level associated with reduced cell numbers in vitro. Finally, we show that targeting GSTCD expression has a robust effect on a modest number of genes including genes that may influence airway cell biology e.g. TGFB1 and IL6 signalling and the more novel finding of a role in adipogenesis. These data provide additional insight into the role of GSTCD in the cell and highlights potential functions in the context of respiratory disease warranting further investigation.

The predicted GST function of GSTCD in part contributed to initial interest in this gene as a candidate/causal gene underlying the association signal with lung function on chromosome 4 as GST enzymes have a detoxification role in the airways [38]. However, investigation of classical GST activity in a GSTCD over-expression system revealed no change in GST activity whereas over-expression of GSTM5 resulted in 2–3 times increased GST expression relative to the negative controls. This finding is intriguing and while we acknowledge the limitation of our study that we did not assess all GST activity, suggests that the assignment of protein family based on initial homology may be in error. Interestingly, GSTCD appears to have only one of the two domains characteristic of the GST family of enzymes [39] (C-terminal α-helical domain) and lacks the N-terminal thioredoxin-type domain. Furthermore, glutathione is thought to bind GST enzymes between the N- and C-terminal domains, therefore indicating that both domains are necessary for GST activity and that GSTCD may simply have GST-like activity [39]. The more thorough structural analyses completed in the current study extends and complements our previous work that suggested GSTCD shares similar motifs with methyltransferase enzymes, GST enzymes and Eukaryotic Elongation Translation Factor 1 epsilon 1 (EEFE1/AIMP3); a component of the aminoacyl tRNA synthase complex [14]. In the current study potential methyl transferase domain was highlighted and warrants further investigation. Methyltransferases have been implicated in cell homeostasis including; e.g. histone methyltransferase (NSD3) which has a role in cell viability and apoptosis [40] and carboxyl methyltranferase acrivity modulates endothelial cell apoptosis [41].

In order to formally assess the potential role of GSTCD in cell homeostasis including cell viability and apoptosis we developed siRNA targeted knockdown in primary human bronchial epithelial cells. This approach effectively reduces GSTCD expression at both the mRNA and protein levels, hence this is a valuable method to study effects of minimal GSTCD levels in a human in vitro model. We then looked at cell proliferation and viability under these conditions. The results suggest a possible role for GSTCD in determining cell number when assessing total cell number which was significantly reduced in cells targeted by GSTCD siRNA. However when proliferation was assessed in parallel these data do not indicate a role in cell proliferation for GSTCD, instead potentially suggesting a role in cell viability. To provide further insight we investigated the effect of targeting GSTCD on apoptosis in these cells. Knockdown of GSTCD did lead to an increase in apoptosis in bronchial epithelial cells albeit this did not reach statistical significance and there was heterogeneity in the data. Interestingly, the increase in apoptosis in cells treated with GSTCD siRNA was more prominent in the siRNA C treated cells which mirrored the more pronounced effects of this siRNA on cell number. However, we cannot exclude that other mechanisms are involved but these data would provide some tentative support for potential link to cell viability and apoptosis.

To provide greater insight into GSTCD function we also completed transcriptomic analyses on primary bronchial epithelial cells treated with two GSTCD siRNA. In these analyses we focussed on genes identified that are differentially expressed following knockdown in both siRNA and across biological replicates which gave a priority list of 17 genes (Table 2). The identification of only 41 genes influenced by knockdown and then the prioritisation to 17 highlights the potentially very limited influence of GSTCD on global gene expression.

Lymphocyte cytosolic protein 1, LCP1 was the most significantly reduced gene when GSTCD is reduced, this gene has been identified by GWAS for non-alcoholic fatty acid liver disease (NFALD) and mRNA from biopsies show a 300% increase in mRNA in this disease compared with control livers [26]. Similarly GPR176 a Gz-linked orphan G-protein coupled receptor which represses cAMP signalling in an agonist-independent manner in the brain [27], had reduced levels which would potentially have a significant effect on cell signalling and is reduced when GSTCD is reduced. The top 6 genes altered by knockdown of GSTCD are also reduced included ETS1, a transcription factor for the activation or repression of numerous genes involved in stem cell development, cell senescence and death and tumorigenesis [42, 43]. AKAP12 a protein which directs the activity of protein kinase A by tethering the enzyme near its physiologic substrates also has reduced expression, it has been shown to mediate barrier functions in fibrotic scarring of the central nervous system [44]. GPD1L a protein found in the cytoplasm associated with the plasma membrane where it binds to the sodium channel SCN5A is however upregulated with a reduction of GSTCD [45]. Interestingly, also in this list of priority genes were genes already linked to respiratory disease including; TGFBR1 and IL6R, two receptor linked to fibrosis and inflammation in the airways respectively [33, 36] see Table 3.

Gene set Enrichment Analysis (GSEA) identified eight pathways that showed significant upregulation with no pathways downregulated (Table 4). A number of these pathways (Adipogenesis, Bile Acid metabolism and Fatty acid metabolism) are involved in fat emulsification, metabolism, storage and removal. The finding that when GSTCD is targeted there are alterations in multiple genes related to adipogenesis (confirmed by both siRNA) is of interest as *GSTCD*^−/−^ mice have been shown to have abnormal body fat amount (International Mouse Phenotyping Consortium). The link between adipose tissue and chronic lung disease is unclear at this time, however there is accumulating evidence for a causative role including adipocyte derived mediators such as leptin in obstructive lung disease and a link between cardiovascular disease and COPD [46]. Again in the *GSTCD*^−/−^ mice there was abnormal electrocardiogram measurement providing a cardiovascular connection in vivo.

The Reactive oxygen species (ROS) pathway being highlighted in the RNA-seq pathway analyses is also of interest with respect to lung disease as Asthma and COPD are characterized by systemic and chronic localized inflammation and oxidative stress. Sources of oxidative stress arise from the increased burden of inhaled oxidants, as well as elevated amounts of ROS released from inflammatory cells. Increased levels of ROS, either directly or via the formation of lipid peroxidation products may play a role in enhancing the inflammatory response in both asthma and COPD (Oxidative stress in asthma and COPD). To specifically address the potential role of GSTCD in ROS pathways again we targeted GSTCD using siRNA, however we were unable to identify a significant effect.

## Conclusion

In conclusion, we provide new insight into the largely uncharacterised protein, GSTCD that is a candidate gene for lung function/COPD. GSTCD has no classical GST activity and protein structure analyses suggest methyltransferase homology. Reduction of GSTCD expression in bronchial epithelial cells by siRNA suggests a role in cell viability. Transcriptomic analyses post GSTCD reduction identified several genes and pathways that may be functionally relevant for the role of GSTCD including genes in pathways implicated in fibrosis and inflammation in the airways. Pathway based approaches identified a role for GSTCD particularly in adipogenesis which complements well *GSTCD*^−/−^ mouse data and warrants further investigation for the potential causative role between GSTCD expression, adipose tissue inflammation and COPD where data already exists for a potential causative link.

## Data Availability

The majority of data generated in this analysis is presented in this published article. The RNA seq datasets are available from corresponding author on reasonable request.

## Abbreviations

C2: Curated gene set 2
CHARGE: Cohorts for Heart and Aging Research in Genomic Epidemiology
COPD: Chronic obstructive pulmonary disease
FDR: False discovery rate
FEV_1_: Forced expiratory volume in one second
FPKM: fragments Per Kilobase of transcript per Million mapped reads
FVC: Forced vital capacity
GSEA: Gene set Enrichment Analysis
GST: Glutathione *S*-transferase
GSTCD: Glutathione *S*-transferase C-terminal domain containing
GWAS: Genome wide association studies
H: Hallmark gene set
HASM: Human airway smooth muscle
HBEC: Human bronchial epithelial cell
IL6: Interleukin 6
I-TASSER: Iterative Threading Assembly Refinement
PDAR: Pre-Developed TaqMan® Assay Reagent
RIN: RNA integrity
ROS: Reactive oxygen species
TGFB: Transforming growth factor β
